# Supplementary material for: The Paf1 complex factors Leo1 and Paf1 promote local histone turnover to modulate chromatin states in fission yeast
Source: EMBO Rep. 2015 Oct 30;16(12):1673–87. doi: 10.15252/embr.201541214 (PMC4687421; doi:10.15252/embr.201541214)
Supplement: Supplementary file 1 — Expanded View Figures PDF [file EMBR-16-1673-s001.pdf]

Expanded View Figures

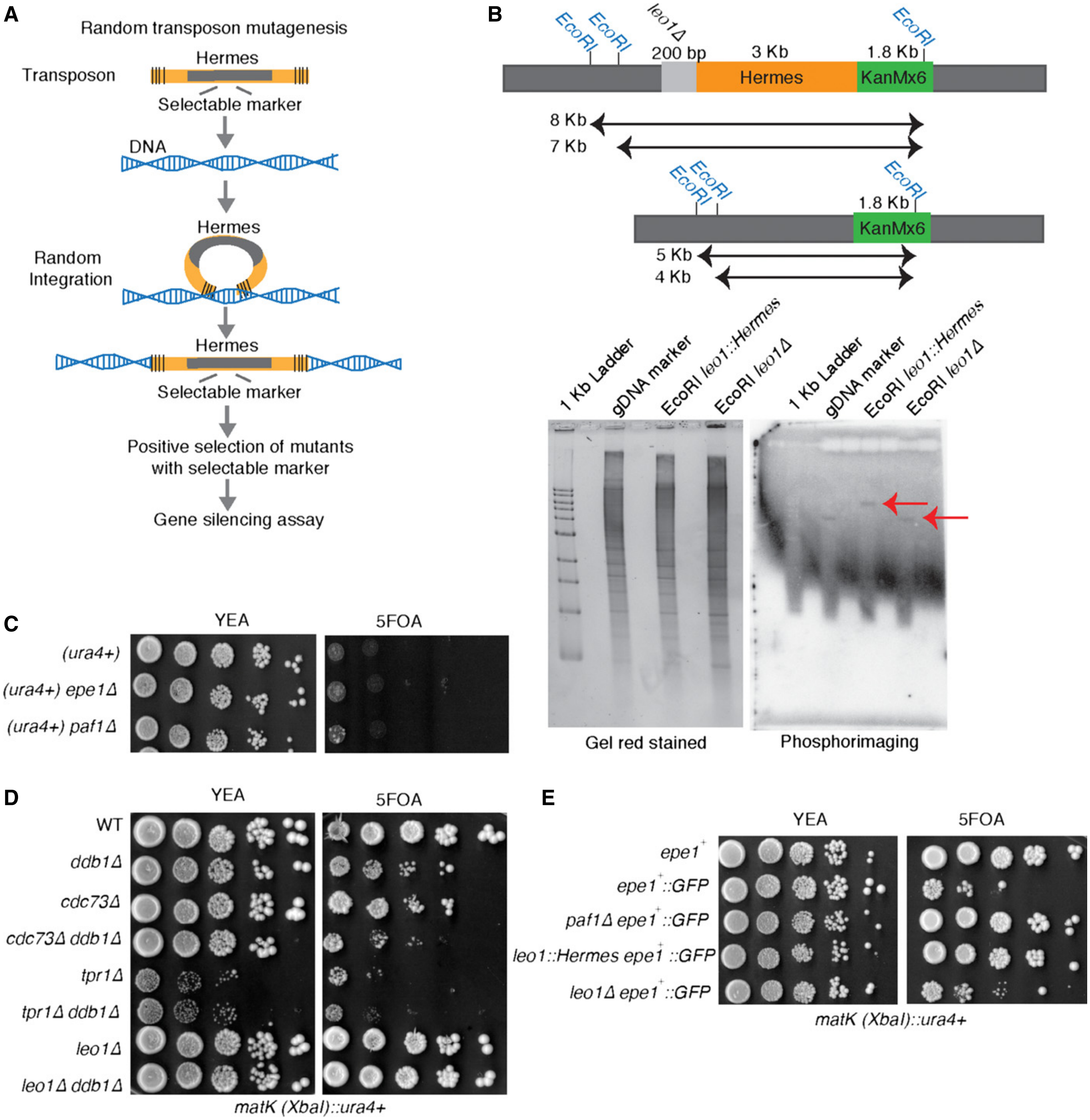

Figure EV1. The transposon mutagenesis system.

A Cartoon of the Hermes transposon screen.

B Southern blot of the *Hermes* strain. Probes were complementary to the kanMX region (top panel) present in both *leo1::Hermes* and *leo1Δ*, leading to a band of length 7–8 kb (*Hermes-kanMX*, lower panel, upper arrow) and a band of length 4–5 kb (*leo1Δ::kanMX*) (lower panel, lower arrow).

C–E The Ddb1 and *epe1::GFP* effect on other components of Paf1C. Genetic interaction between *ddb1* and *cdc73* and *tpr1.* (C) Spotting assay with *ura4*$^{+}$ at its endogenous locus. (D) Spotting assay with strains in *ddb1Δ* background. (E) Spotting assay in *epe1*$^{+}$*::GFP*-derived strains.

 © 2015 The Authors

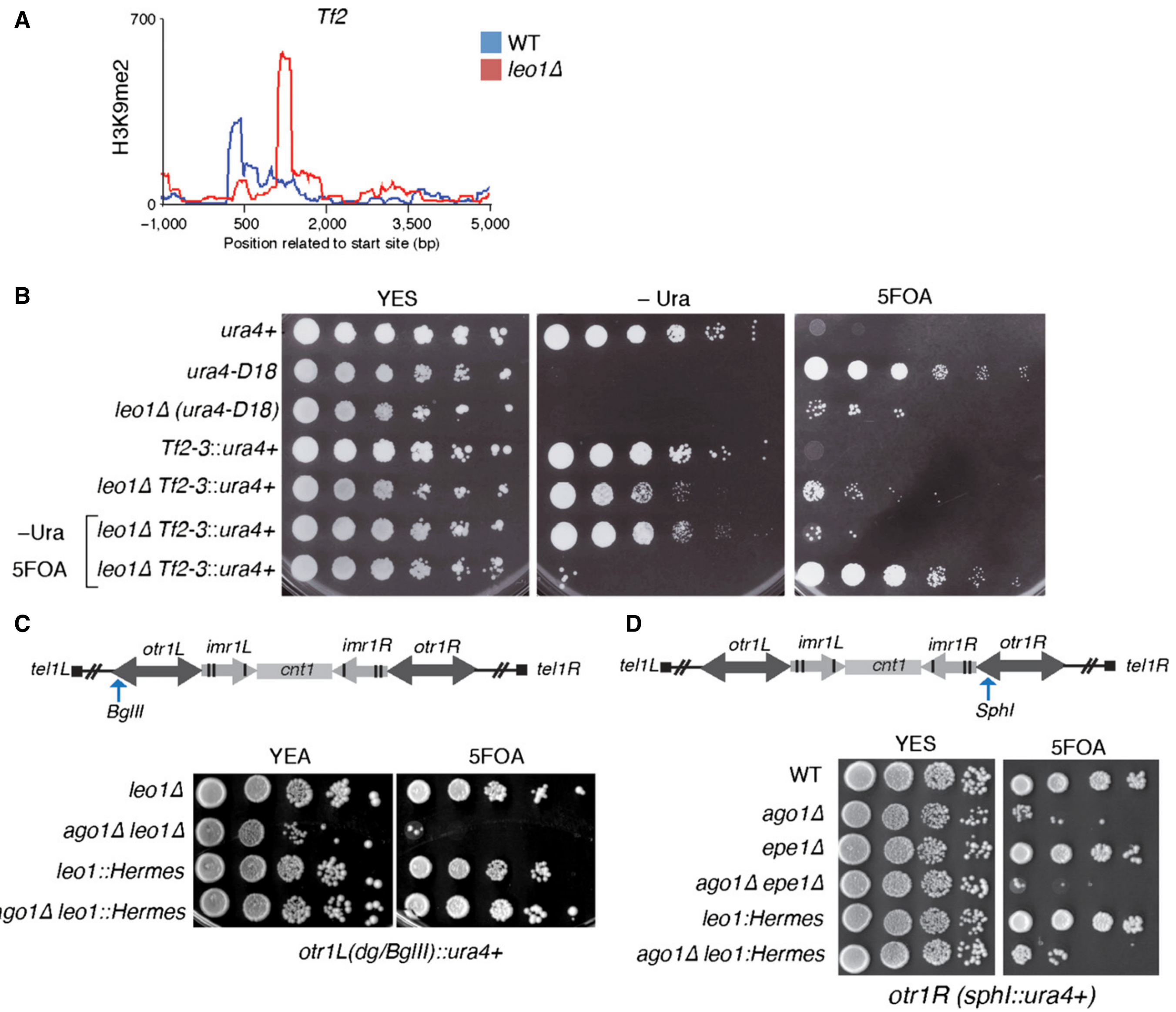

Figure EV2. Heterochromatin formation over retrotransposable element *Tf2* pericentric region of chromosome 1.

A ChIP–exo for H3K9me2 at Tf2.

B Position variegation at Tf2 effect by *leo1*Δ. Spotting assay on –Ura and FOA plates of strains previously grown on –Ura or FOA plates.

C Spotting assay of indicated strains on YEA and 5FOA media with *ura4*$^{+}$ integrated at the left pericentric region of chromosome 1.

D Spotting assay of indicated strains on YEA and 5FOA media with *ura4*$^{+}$ integrated at the right pericentric region of chromosome 1.

© 2015 The Authors

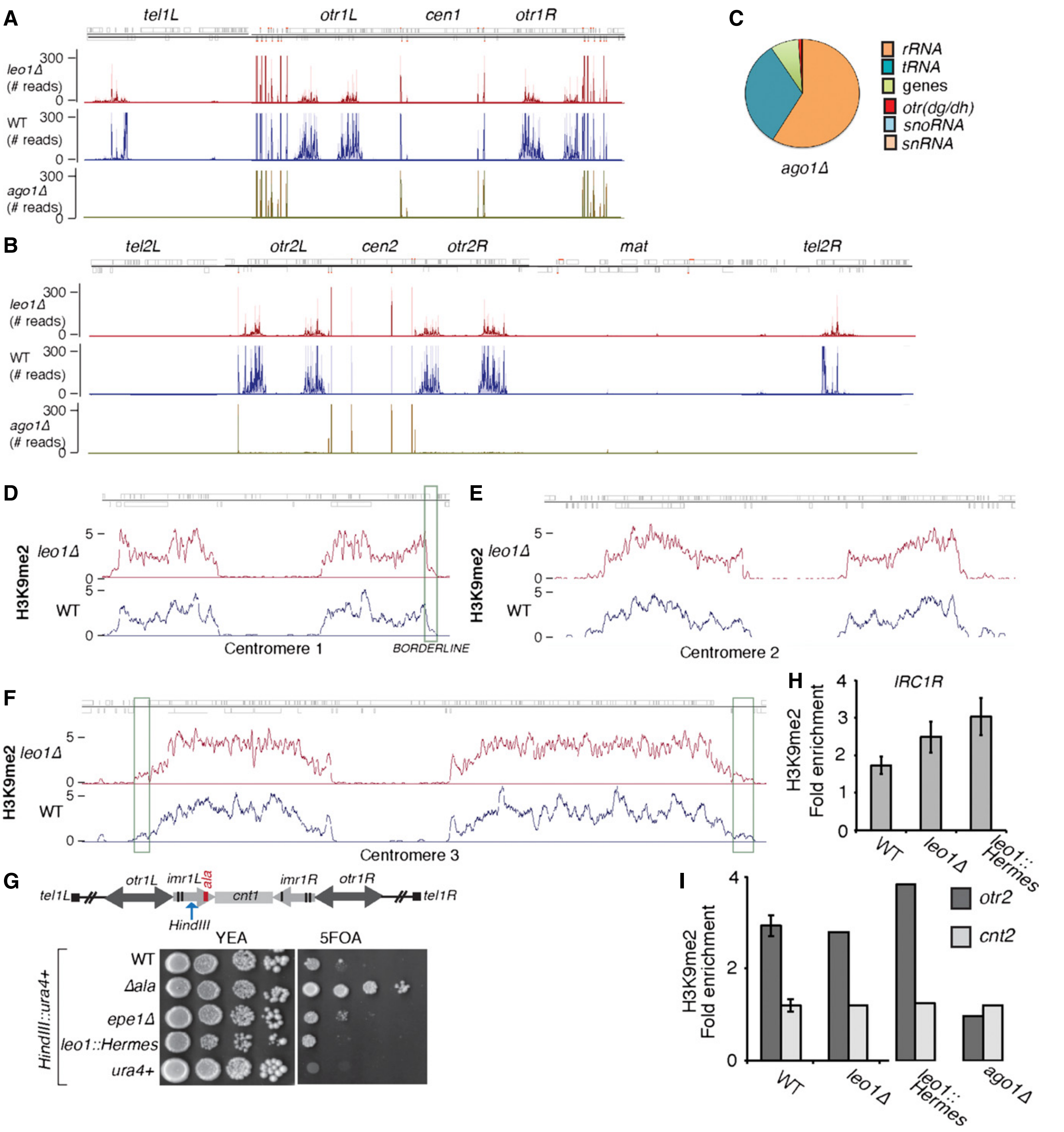

Figure EV3. Small RNAs and heterochromatin at telomeres and centromeres.

A sRNA distribution at *tel1L* and the centromere of chromosome 1.

B sRNA distribution at *tel2L*, the centromere of chromosome 2, the *mat* region, and *tel2R*.

C Distribution of sRNA populations in *ago1*Δ.

D–F H3K9me2 ChIP–exo over centromere 1 (D), centromere 2 (E), and centromere 3 (F). Green boxes indicate the location of the BORDERLINE transcript (Keller *et al*, 2013) and the IRC3 elements (Buhler *et al*, 2008).

G Spotting of strains with *ura4*$^{+}$ at the euchromatic (inner centromeric) side of tRNA barrier at *cen1R*.

H, I H3K9me2 ChIP–qPCR over the *IRC1R* locus (H) and the outer repeats (*otr2*) and central core (*cnt2*) of centromere 2 (I). Experiments were done in three independent experiments; error bars show the SD.

Data information: (A–F) Two independent experiments were performed, and the profiles show the average.

 © 2015 The Authors

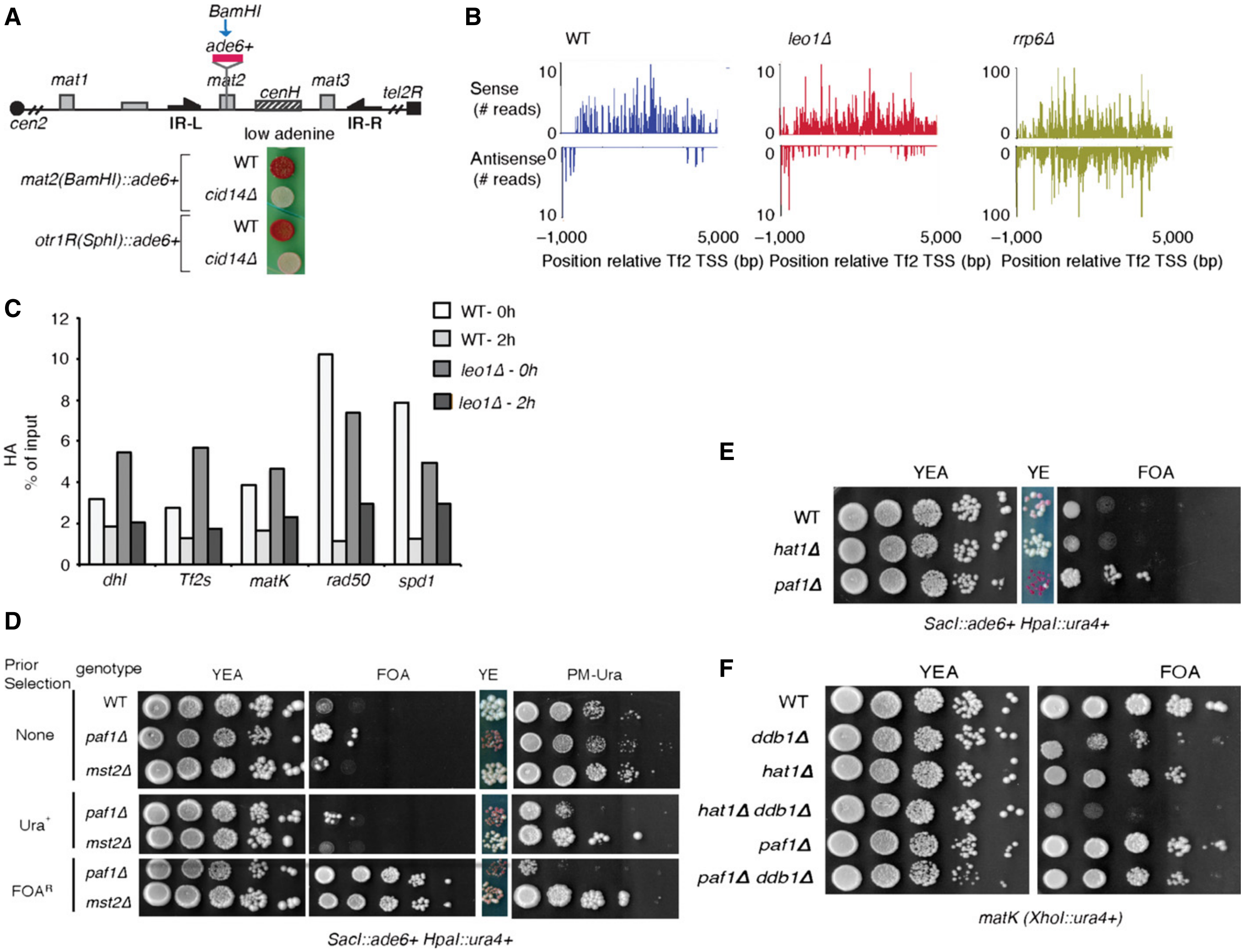

Figure EV4. Effect of strain genotype on gene expression (or histone retention) at various chromosomal locations.

A, B Retrotransposable element Tf2 is silenced by an siRNA-independent mechanism in *leo1*Δ. (A) Growth of indicated strains, with *ade6*$^{+}$ integrated at two locations, on a low-adenine plate. (B) Distributions of sRNA mapping to the 13 Tf2 retrotransposons. Normalized reads are aligned relative to the transcription start site, sense (top panel) and anti-sense direction (bottom panel) in WT (blue), *leo1*Δ (red), and *rrp6*Δ (green) cells.

C, D Histone turnover and the effects of acetyltransferases on heterochromatin formation. (C) ChIP–qPCR using antibody against HA in cells with the RITE construct, using primers for *dh1*, *tf2*, *matK*, *rad50*$^{+}$, and *spd1*$^{+}$. ChIP was performed in two independent experiments. (D) Position variegation effect by Mst2 and Paf1. Spotting assay of strains preselected as being Ura^{+} or FOA^{R} as indicated.

E Spotting assay of strains with *ura4*$^{+}$ integrated at *matL*, and *ade6*$^{+}$ at *IR-L*.

F Spotting assay of strains with *ura4*$^{+}$ integrated at *matK*.

© 2015 The Authors

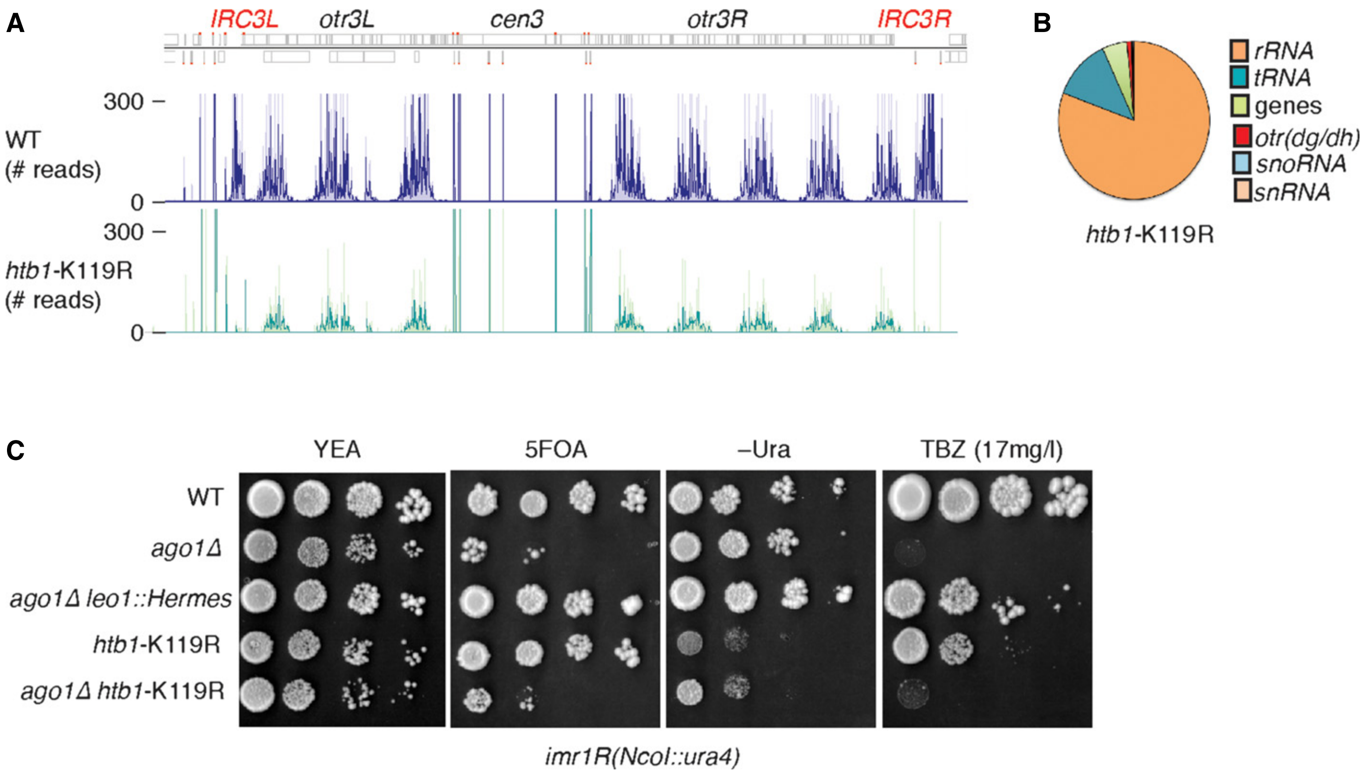

Figure EV5. The contribution of H2Bub1 on silencing of the pericentric chromatin.

A sRNA profile over centromere 3. The number of reads from two independent experiments was normalized to reads mapping to tRNAs. Red dots in top panel indicate positions of tRNAs.

B sRNA profile in *htb1*-K119R.

C Spotting assay of strains with *ura4*$^{+}$ integrated at *imr1R*.

 © 2015 The Authors
