# Supplementary material for: The Paf1 complex factors Leo1 and Paf1 promote local histone turnover to modulate chromatin states in fission yeast
Source: EMBO Rep. 2015 Oct 30;16(12):1673–87. doi: 10.15252/embr.201541214 (PMC4687421; doi:10.15252/embr.201541214)
Supplement: Supplementary file 2 — Table EV1 [file EMBR-16-1673-s002.docx]

**Table EV1:** Strains used in this study

| **Strain** | **Genotype** | **Figure** |
| --- | --- | --- |
| AP260 | *mat1-M-smt0 (SacI)::ade6+ (HpaI)::ura4+ ade6-210 ura4-D18 leu1-32 his2* | 1.B, 2.E, 2.F,7.E |
| AP3272 | *mat1-M-smt0 (SacI)::ade6+ (HpaI)::ura4+ ade6-210 ura4-D18 (or DS/E) leu1-32 his2 epe1∆::hygMX* | 1.B, 2.C, 2.E, 2.F |
| AP2647 | *mat1-M-smt0 (SacI)::ade6+ (HpaI)::ura4+ ade6-210 ura4-D18 leu1-32 his2 leo1∆::kanMX6 (Hermes)* | 1.B, 1.E, 1.F, 2.C, 2.E, 2.F  5.A,5.B, EV6.E, EV6.F |
| AP3246 | *mat1-M-smt0 (SacI)::ade6+ (HpaI)::ura4+ ade6-210 ura4-D18 leu1-32 his2 leo1Δ::kanMX* | 1.B, 2.B |
| Hu2640 | *h+ ade6-M216 leu1-32 ura4 DS/E otrR(Sph1)::ura htb1-K119R:: kanMX* | 2.D |
| Hu303 | *972 h-* | 1.C, 1.E, 1.F, 2.D, 3.B  3.B, 3,C, 4.D, 4.A, 4.B, 4.C,  4.D, 4.E, 6.A, 6.B, EV3.A,  EV3.B, EV5.A, EV5.B, EV6.A,  EV6.B, EV6.C, EV6.E, EV6.F, EV7.B, EV9.A |
| Hu2799 | *leo1:HA::kanMX6* | 1.C, 1.E, 1.F, 4.A, 4.B,  4.C, 4.D |
| Hu2800 | *leo1(1-169):HA::kanMX6* | 1.C, 1.E, 1.F |
| Hu2689 | *h+ ade6-M216 leu1-32 ura4-D18 leo1∆::kanMX* | 1.C, 1.E, 1.F, 2.D, 3.B, 3.C  4.A, 4.B, 4.C,4.E, 6.A, 6.B, EV3.B, EV5.A, EV5.B,EV6.A, EV6.B, EV6.C, EV6.E,EV6.F, EV7.B |
| AP2757 | *mat1-M-smt0 (SacI)::ade6+ (HpaI)::ura4+ ade6-210 ura4-D18 leu1-32 his2 paf1Δ::kanMX* | 2.B, 2.C, 2.E, 2.F, 7.E |
| AP2755 | *mat1-M-smt0 (SacI)::ade6+ (HpaI)::ura4+ ade6-216 ura4-D18 leu1-32 his2 tpr1Δ::kanMX* | 2.B |
| AP3290 | *mat1-M-smt0 (SacI)::ade6+ (HpaI)::ura4+ ade6-210 or 216 ura4-D18 leu1-32 his2 cdc73Δ::kanMX* | 2.B |
| AP2851 | *mat1-M-smt0 (SacI)::ade6+ (HpaI)::ura4+ ade6-210? ura4-D18 leu1-32 his2 htb1-K119R::kanMX* | 2.E, 2.F |
| AP2805 | *mat1-M-smt0 (SacI)::ade6+ (HpaI)::ura4+ ade6-210 ura4-DS/E leu1-32 his2 shf1∆::kanMX* | 2.E, 2.F |
| AP2801 | *mat1-M-smt0 (SacI)::ade6+ (HpaI)::ura4+ ade6-210 ura4-DS/E leu1-32 his2 rfp1∆::kanMX* | 2.E, 2.F |
| AP2736 | *mat1-M-smt0 (SacI)::ade6+ (HpaI)::ura4+ ade6-210 ura4-D18 leu1-32 his2 set1∆::KanMX* | 2.E, 2.F |
| AP3323 | *mat1-M-smt0 (BglII)::ade6+ (Xba1):: ura4+ ade6-210 ura4-D18 leu1-32 spd1Δ:hygMX ddb1Δ::natMX* | 3.A, 3.B, 3.C, 4.C, EV2.B |
| AP3347 | *mat1-M-smt0 (BglII)::ade6+ (Xba1):: ura4+ ade6-210 ura4-D18 leu1-32 spd1Δ:hygMX ddb1Δ::natMX leo1Δ::kanMX* | 3.A, 3.B, 3.C, 4.C, EV2.B |
| AP3349 | *mat1-M-smt0 (BglII)::ade6+ (Xba1):: ura4+ ade6-210 ura4-D18 leu1-32 leo1Δ::KanMX* | 3.A, 3.B, 3.C |
| AP3355 | *mat1-M-smt0 (BglII)::ade6+ (Xba1):: ura4+ ade6-210 or 216 ura4-D18 leu1-32 paf1Δ::kanMX* | 3.A |
| AP3353 | *mat1-M-smt0 (BglII)::ade6+ (Xba1):: ura4+ ade6-210 or 216 ura4-D18 leu1-32 spd1Δ:hygMX ddb1Δ::natMX paf1Δ::kanMX* | 3.A |
| Hu29 | *h- ade6-M210 leu1-32 ura4-D18* | 4.E, EV3.B |
| Hu2703 | *h- ade6-210 ura4-D18 leu1-32 ura4 before ATG of Tf2-3 (PEY914)* | 4.E, EV3.B |
| Hu2824 | *h- ade6-210 ura4-D18 leu1-32 ura4 before ATG of Tf2-3 leo1∆::kanMX* | 4.E, EV3.B |
| AP2937 | *mat1-M-smt0 (SacI)::ade6+ ade6- 210 ura4-D18 leu1-32 his2- SPBAC23H3.14::ura4+ epe1∆::kanMX* | 4.F |
| AP2950 | *mat1-M-smt0 (SacI)::ade6+ ade6- 210 ura4-D18 leu1-32 his2- SPBAC23H3.14::ura4+* | 4.F, 4.G |
| AP2939 | *mat1-M-smt0 (SacI)::ade6+ ade6- 210 ura4-D18 leu1-32 his2- SPBAC23H3.14::ura4+ leo1::kanMX (Hermes)* | 4.F, 4.G |
| AP3349 | *mat1-M-smt0 (SacI)::ade6+ ade6- 210 ura4-D18 leu1-32 his2- SPBAC23H3.14::ura4+ paf1∆::kanMX* | 4.F, 4.G |
| AP167 | *mat1-M-smt0 (BglII)::ade6+ (Xba1):: ura4+ ade6-210 ura4-D18 leu1-32* | 5.C, EV2.A |
| AP3364 | *mat1-M-smt0 (BglII)::ade6+ (Xba1):: ura4+ ade6-210 or 216 ura4-D18 leu1-32 epe1+::GFP(venus)LEU2* | 5.C |
| AP2727 | *mat1-M-smt0 (SacI)::ade6+ ade6- 210 ura4-D18 or DS/E leu1-32 his2- imrL (dg-glu) NcoI::ura4* | 5.C, 5.D, 5.E, 6.C |
| AP3447 | *mat1-M-smt0 (BglII)::ade6+ (Xba1):: ura4+ ade6-210 or 216 ura4-D18 leu1-32 epe1+::GFP(venus)LEU2 leo1::kanMX6 (Hermes)* | 5.C, EV2.C |
| AP3013 | *mat1-M-smt0 (SacI)::ade6+ ade6- 210 ura4- DS/E leu1-32 his2- imrL (dg-glu) NcoI::ura4 epe1∆:kanMX6* | 5.C |
| AP3017 | *mat1-M-smt0 (SacI)::ade6+ ade6- 210 ura4- DS/E leu1-32 his2- imrL (dg-glu) NcoI::ura4 ago1∆::natMX* | 5.C, 5.E |
| AP3019 | *mat1-M-smt0 (SacI)::ade6+ ade6- 210 ura4-D18 or DS/E leu1-32 his2- imrL (dg-glu) NcoI::ura4 leo1::kanMX6 (Hermes) ago1∆::natMX* | 5.A, 5.B, 5.C, 5.E |
| AP1678 | *mat1-Msmt0 mat2(BamHI)::ade6+ otr1(dh/BglII)::ura4+ leu1-32 his2 ura4-DS/E ade6-210* | 5.C |
| AP3105 | *mat1-Msmt0 mat2(BamHI)::ade6+ otr1(dh/BglII)::ura4+ leu1-32 his2 ura4-DS/E or D18 ade6-210 ago1∆::natMX* | 5.C |
| AP3109 | *mat1-Msmt0 mat2(BamHI)::ade6+ otr1(dh/BglII)::ura4+ leu1-32 his2 ura4-DS/E or D18 ade6-210 or DN/N epe1∆::kanMX* | 5.C |
| AP3107 | *mat1-Msmt0 mat2(BamHI)::ade6+ otr1(dh/BglII)::ura4+ leu1-32 his2 ura4-DS/E or D18 ade6-210 or DN/N ago1::natMX epe1∆::kanMX* | 5.C |
| AP2722 | *mat1-Msmt0 mat2(BamHI)::ade6+ otr1(dh/BglII)::ura4+ leu1-32 his2 ura4-DS/E ade6-210 leo1::kanMX6 (Hermes)* | 5.C |
| AP3103 | *mat1-Msmt0 mat2(BamHI)::ade6+ otr1(dh/BglII)::ura4+ leu1-32 his2 ura4-DS/E or D18 ade6-210 ago1::natMX leo1::kanMX6 (Hermes)* | 5.C |
| AP2727 | *mat1-M-smt0 (SacI)::ade6+ ade6- 210 ura4-D18 or DS/E leu1-32 his2- imrL (dg-glu)NcoI::ura4* | 5.C, 5.D, 5.E |
| AP3017 | *mat1-M-smt0 (SacI)::ade6+ ade6- 210 ura4- DS/E leu1-32 his2- imrL (dg-glu)NcoI::ura4 ago1∆::natMX* | 5.C |
| AP3013 | *mat1-M-smt0 (SacI)::ade6+ ade6- 210 ura4- DS/E leu1-32 his2- imrL (dg-glu)NcoI::ura4 epe1∆:KanMX6* | 5.C |
| AP3015 | *mat1-M-smt0 (SacI)::ade6+ ade6- 210 ura4-D18 or DS/E leu1-32 his2- imrL (dg-glu)NcoI::ura4 epe1∆:kanMX6 ago1∆::natMX* | 5.C |
| AP2728 | *mat1-M-smt0 (SacI)::ade6+ ade6- 210 ura4-D18 leu1-32 his2- imrL (dg-glu)NcoI::ura4 leo1::kanMX6 (Hermes)* | 5.C |
| AP3019 | *mat1-M-smt0 (SacI)::ade6+ ade6- 210 ura4-D18 or DS/E leu1-32 his2- imrL (dg-glu)NcoI::ura4 leo1::kanMX6 (Hermes) ago1∆::natMX* | 5.C |
| AP2867 | *mat1-M-smt0 (SacI)::ade6+ ade6-210 or DN/N ura4-D18 leu1-32 his2- imrL (dg-glu)NcoI::ura4 pob3∆::natMX6* | 5.E |
| AP3153 | *mat1-M-smt0 (SacI)::ade6+ ade6-216 ura4-D18 or DS/E leu1-32 his2- imrL (dg-glu)NcoI::ura4 cid14Δ::natMX* | 6.C |
| AP2728 | *mat1-M-smt0 (SacI)::ade6+ ade6- 210 ura4-D18 leu1-32 his2- imrL (dg-glu)NcoI::ura4 leo1::kanMX6 (Hermes)* | 6.C |
| AP3149 | *mat1-M-smt0 (SacI)::ade6+ ade6- 210 ura4-D18 leu1-32 his2- imrL (dg-glu)NcoI::ura4 leo1::kanMX6 (Hermes) cid14∆::natMX* | 6.C |
| AP3445 | *mat1-Msmt0 mat2(BamHI)::ade6+ otr1(dh/BglII)::ura4+ leu1-32 his2 ura4-DS/E or D18 ade6-210 or DN/N ars1::prad15 cre-EBD-leu2+ H3.2-lox-HA- hygR-lox-T7 his3-D1(?) arg3-D4 (?)* | 7.B, 7.C, EV8.A |
| AP3446 | *mat1-Msmt0 mat2(BamHI)::ade6+ otr1(dh/BglII)::ura4+ leu1-32 his2 ura4-DS/E or D18 ade6-210 leo1::KanMX6 ars1::prad15 cre-EBD-leu2+ H3.2-lox-HA- hygR-lox-T7 his3-D1? arg3-D4 (?)* | 7.B, 7.C, EV8.A |
| AP3433 | *mat1-Msmt0 (BglII)::ade6+ (XbaI)::ura4 leu1-32 his2 ura4-DS/E or D18 ade6-210 leo1∆::kanMX6 ddb1∆::natMX ars1::prad15 cre-EBD-leu2+ H3.2-lox-HA- hygR-lox-T7 his3-D1? arg3-D4 (?)* | 7.B,7.C, EV8.A |
| AP3922 | *mat1Msmt0 (BglII)::ade6+ (Xba1):: ura4+  ade6-210 or 216  ura4-D18  leu1-32 ars1::prad15 cre-EBD-leu2+ H3.2-lox-HA-hygR-lox-T7 arg3-D4(?) mst2∆::kanMX4* | 7.D |
| AP3921 | *mat1Msmt0 (BglII)::ade6+ (Xba1):: ura4+  ade6-210 or 216  ura4-D18  leu1-32 ddb1Δ::natM ars1::prad15 cre-EBD-leu2+ H3.2-lox-HA- hygR-lox-T7 arg3-D4? mst2∆::kanMX* | 7.D |
| AP167 | *mat1Msmt0  (BglII)::ade6+ (Xba1):: ura4+  ade6-210  ura4-D18  leu1-32* | 7.D |
| AP3434 | *mat1Msmt0 (BglII)::ade6+ (Xba1):: ura4+  ade6-210  ura4-D18  leu1-32 ddb1Δ::natMX ars1::prad15 cre-EBD-leu2+ H3.2-lox-HA- hygR-lox-T7 arg3-D4* | 7.D |
| AP3360 | *mat1-M-smt0 (BglII)::ade6+ (Xba1):: ura4+ ade6-210 or 216 ura4-D18 leu1-32 paf1Δ::kanMX4 epe1+::GFP(venus)LEU2* | EV2.A |
| AP3388 | *mat1-M-smt0 (BglII)::ade6+ (Xba1):: ura4+ ade6-210 or 216 ura4-D18 leu1-32 epe1+::GFP(venus)LEU2 leo1Δ::kanMX* | EV2.C |
| AP3379 | *mat1-M-smt0 (BglII)::ade6+ (Xba1):: ura4+ ade6-210 or 216 ura4-D18 leu1-32 cdc73Δ::kanMX* | EV2.B |
| AP3370 | *mat1-M-smt0(BglII)::ade6+ (Xba1):: ura4+ ade6-210 or 216 ura4-D18 leu1-32 spd1Δ:hygMX ddb1Δ::natM cdc73Δ::kanMX* | EV2.B |
| AP3384 | *mat1-M-smt0 (BglII)::ade6+ (Xba1):: ura4+ ade6-210 or 216 ura4-D18 leu1-32 tpr1Δ::kanMX* | EV2.B |
| AP3382 | *mat1-M-smt0 (BglII)::ade6+ (Xba1):: ura4+ ade6-210 or 216 ura4-D18 leu1-32 spd1Δ:hygMX ddb1Δ::natMX tpr1Δ::kanMX* | EV2.B |
| AP2791 | *mat1-M-smt0 (SacI)::ade6+ ade6- 210 ura4- DS/E otr1R SphI::ura4 leu1-32 his2-* | EV4.B |
| AP2955 | *mat1-M-smt0 (SacI)::ade6+ ade6- 210/DN/N ura4-DS/E leu1-32 his2- otr1R SphI::ura4+ ago1∆::natMX* | EV4.B |
| AP2895 | *mat1-M-smt0 (SacI)::ade6+ ade6- 210/216/DN/N ura4-DS/E leu1-32 his2- otr1R SphI::ura4+ epe1∆::kanMX* | EV4.B |
| AP2952 | *mat1-M-smt0 (SacI)::ade6+ ade6- 210/DN/N ura4-DS/E or D18 leu1-32 his2- otr1R SphI::ura4+ epe1∆::kanMX ago1∆::natMX* | EV4.B |
| AP2971 | *mat1-M-smt0 (SacI)::ade6+ ade6- 210/DN/N ura4-D18 leu1-32 his2- otr1R SphI::ura4+ leo1::kanMX (Hermes)* | EV4.B |
| AP2961 | *mat1-M-smt0 (SacI)::ade6+ ade6- 210/DN/N ura4-DS/E leu1-32 his2- otr1R SphI::ura4+ ago1∆::natMX leo1::kanMX (Hermes)* | EV4.B |
| Hu1480 | *h- ago1∆::kanMX* | EV5.A, EV5.B |
| AP3137 | *mat1-M-smt0 (SacI)::ade6+ ade6- 210 ura4-D18 or DS/E leu1-32 his2- imrL (ClaI-EcoRI) HinDIII::ura4 oriI* | EV6.D |
| AP3135 | *mat1-M-smt0 (SacI)::ade6+ ade6- 210 ura4-D18 leu1-32 his2- imr1R::HindIII)Δala ura4+ oriI* | EV6.D |
| AP3155 | *mat1-M-smt0 (SacI)::ade6+ ade6- 210 ura4-D18 or DS/E leu1-32 his2- imrL (ClaI-EcoRI) HinDIII::ura4 oriI epe1∆::kanMX6* | EV6.D |
| AP3157 | *mat1-M-smt0 (SacI)::ade6+ ade6- 210 ura4-D18 or DS/E leu1-32 his2- imrL (ClaI-EcoRI) HinDIII::ura4 oriI leo1::kanMX6 (Hermes)* | EV6.D |
| Hu2464 | *h- ade6-704 ura4 leu1-32 rrp6∆::kanMX6* | EV7.B |
| AP333 | *mat1Msmt0 BamHI::ade6-R*  his2 *ura4-D18 ade6-210 leu1-32* | EV7.A |
| AP3232 | *mat1Msmt0 mat2(BamHI)::ade6+ his2 ura4-D18 ade6-210 leu1-32 cid14∆::natMX* | EV7.A |
| AP3236 | *mat1Msmt0 ade6-210 ura4-D18 leu1-32 his2- otr1R (dg-glu) SphI::ade6+* | EV7.A |
| AP3238 | *mat1Msmt0 ade6-210 ura4-D18 leu1-32 his2- cid14D::natR otr1R (dg-glu) SphI::ade6+* | EV7.A |
| AP3971 | *mat1Msmt0 (BglII)::ade6+ (Xba1):: ura4+  ade6-210  ura4-D18  leu1-32 ddb1Δ::natMX hat1Δ::kanMX ars1::prad15 cre-EBD-leu2+ H3.2-lox-HA- hygR-lox-T7 arg3-D4* | EV8.D |
| AP2727 | *mat1-M-smt0 (SacI)::ade6+ ade6- 210 ura4-D18 or DS/E leu1-32 his2- imrL (dg-glu)NcoI::ura4* | EV9.C |
| AP3017 | *mat1-M-smt0 (SacI)::ade6+ ade6- 210 ura4- DS/E leu1-32 his2- imrL (dg-glu)NcoI::ura4 ago1∆::natMX* | EV9.C |
| AP3019 | *mat1-M-smt0 (SacI)::ade6+ ade6- 210 ura4-D18 or DS/E leu1-32 his2- imrL (dg-glu)NcoI::ura4 leo1∆::kanMX6 ago1∆::natMX* | EV9.C |
| AP3087 | *mat1Msmt0 (SacI)::ade6+ ade6- 210 ura4-D18 leu1-32 his2- imrL (dg-glu)NcoI::ura4 htb1-K119R::kanMX* | EV9.C |
| AP3085 | *mat1Msmt0 (SacI)::ade6+ ade6- 210 ura4- DS/E leu1-32 his2- imrL (dg-glu)NcoI::ura4 ago1∆::natMX htb1-K119R::kanMX* | EV9.C |
