# Supplementary material for: The Paf1 complex factors Leo1 and Paf1 promote local histone turnover to modulate chromatin states in fission yeast
Source: EMBO Rep. 2015 Oct 30;16(12):1673–87. doi: 10.15252/embr.201541214 (PMC4687421; doi:10.15252/embr.201541214)
Supplement: Supplementary file 3 — Table EV2 [file EMBR-16-1673-s003.docx]

**Table EV2:** Primers used in this study

| Primer name | Sequence |
| --- | --- |
| HpaI_TM_F: | AGTTCAAGGTATAGTCCGATCAAGA |
| HpaI_TM_R: | GCTGGACCACTATGAGAAGTGA |
| SacI_TM_F: | CAATGATTAATATGAGAGCTCCAGG |
| SacI_TM_R: | GATGTAATCATTATAGCCCATGCAA |
| matK-F | TAGTATTCTGTCGAAATTATCGAAAGCTA |
| matK-R | GAAACTGAAGCAGGGAAAAATGTAG |
| Cnt2-F | CCGACTCAGTTGACGTTACCTTT |
| Cnt2-R | TGTCCACCACAAATAGTTCAGCAT |
| Otr2-F | CGCTTCTCCTTAATCCATTTGTGT |
| Otr2-R | GGTGGATGGTGGACGCTTT |
| Tf2-F | GGTAGGCAGTTTATGTGCTC |
| Tf2-R | AGAACAGCCTCGTATGGTAA |
| IRC1-F: | GCT AAA TCA AGA CTG TGA TGG TGT |
| IRC1-R: | ACT CAA TCC GTG GAC GTA TCA ACT |
| tRNA_F: | AAG ATT CTA AGG AAG TAA GCC AAG |
| tRNA_R: | TAC CTC TTC ATC CAA GCA TCG |
| dh1-F | TGGAACCGGCGATTGAGA |
| dh1-R | CTTCCTTTGGGCTAAAATCATTGT |
| Rad50-F | AAGGATTTCAAAGCGTGAGC |
| Rad50-R | GAAGGGATCATGACAGAGGTG |
| Spd1-F | GCAAGCGAGTTATGACCACAAA |
| Spd1-R | GCTGAGGACGCATTGAAGACT |
| Pyk1-F | GCG ACG AAA ACT TGA GAG GC |
| Pyk1-R | CTT CCT TCA TCC GTC GTG CT |
| Scm3-F | GTT GAT GCT TCA GCT TTG GA |
| Scm3-R | TGT ATC CAA ACC ACG CTG AT |
| SB-F | CGGTTTGGTTGATGCGAGTG |
| SB-R | ACAGCAGTATAGCGACCAGC |
